# Warming Houses

**Published:** 1872-06

**Authors:** 


### WARMING HOUSES,

As ordinarily practiced, gives twice as much heat at the ceiling, where it is not wanted, and half as much on the floor, where it is wanted, thus absolutely reversing the wants of the body; for it has been accepted for ages, that the head should by all means be kept cool and the feet warm, without which latter, there cannot be good health under any circumstances; in addition, the moisture of the room from vessels of liquid and other soft substances, and the impurities of the air which come from the lungs at each breath, being heavier than pure air, settle about the lower part of the room, thus giving the occupants the worst air of the room to breathe, causing it to enter and poison the circulation, and at every respiration increasing the impurity of the blood.

There is always a current of air in a room where there is fire, from the ceiling to the floor, for two reasons; first, when confined, hot air tends to dispel the cold; hot air has more power than frost. Second, the hot air at the ceiling becomes impregnated with gas and moisture, becomes heavy, and falls to the floor. Third, there is a current of air formed by these combined circumstances, with the aid of the additional fact that as the cold air comes in under the door and windows of a winter's day, it begins to get warmer in a moment, and as it warms, it expands, is rarefied, and rises, aided by the constantly incoming cold air; for hot air does not necessarily rise of itself, unless there is something to give it a start; besides, heated air absorbs moisture, and this makes it heavier; so as to a room, the cold air is heavier, and crowds the lighter, warmer air out of its place, unless something takes place to make this warmer air heaver than the colder, as by the absorption of moisture; then it falls into the cold.

For this reason, opening a door which leads into a cold bedroom, from a warm family room, results in transferring the warm air, loaded with the impurities above named to the cold walls, and bedding, and windows, because a particle of warm air with the contained moisture, will not only settle on level cold surfaces, but will settle against the sides of perpendicular cold surfaces, as witness the "frost" on the window panes of our chambers, when we wake up in the morning, the weather having become excessively cold during the night, making the glass colder than the breath.

The fact is, the door leading from a family room to a chamber had better not be opened at all, for every time it is opened more or less of the warm air, loaded as above, will be deposited on the walls and other surfaces, setting up fungus growths which are thus fed, and by which they grow, and give off fumes which poison the air most thoroughly.

And yet many persons will remember that in former times the door opening from the family room to a sleeping-room was thought to send warmth into it, and also to be a means of promoting the circulation of the air. It is reported that a whole family who had such an arrangement during an entire winter had been thereby thrown into a miserable state of health.

The best and most frequently available way of

VENTILATING A CHAMBER, both winter and summer, is to hoist a window that leads to all out doors, and let the air be driven out by a draft from the inside of the house, or have the fire-place kept always open, and make a draught from a window or door towards it.

It is therefore better that the door between a warm sitting-room and a cold chamber should not be opened at all, or should be always kept open, and a door or window of the chamber should be kept open an hour or two every day in order to compel a draught of air, which shall carry out all the contained impurities, to be replaced by the fresher out-door air.

After all, with our present habits and views, acting up to the knowledge we have, waiting meanwhile for the developments which are constantly made by scientific investigation, one fact is clear and indisputable, that the family room of every house should be the largest in it, should have the most windows, should face the south, so that the drying, warming, cheering sunshine, should always flood the room with light, and life, and gladness.

In addition there should be a large fire-place kept liberally supplied with wood from October to April; by this means, every time the door is opened a liberal supply of pure cold air comes in with a rush, direct to the fire-place, sweeping into it and up the chimney all the impure air and noxious gases and odors which, as above shown, are in the lower part of the room.

It can scarcely be denied that this homely method of warming and ventilating a room is the most perfect, the most comfortable, and the most healthful ever proposed in modern times; no other method will compare with it for a moment. In some places it is too expensive, in others it is impossible; but there is a substitute, not only as good but much more available, because in very cold weather it keeps one person pretty busy to keep one fire burning, if the wood has to be sawed by hand or chopped; this substitute costs from fifteen to fifty dollars, according to style and finish, and will last two or three lifetimes; it is Dixon's (of Philadelphia) low down grate, which will burn wood, peat, soft coal, or hard coal, and kindle these latter without a blower. The coal is put flat on the hearth, is supplied with air from out-doors, and is beyond all question the most perfect, economical, healthful method known of warming single rooms; and there is no hesitation in saying that every house where there is a family of intelligence, cultivation, and refinement, should have at least one such room-warmer in the house, and that should be the family room, the room where most of the time is spent, consequently most needing a pure, warm, and cheerful atmosphere. There is scarcely a single professional man of any position in the whole of that city, occupying his own house, who has not at least one of these most delightful contrivances; it can be placed in the stead of an ordinary grate, in half a day, and as at this season multitudes are either building, or contemplating building houses for themselves, the suggestions are timely.

